# Looking Back, Leaning Forward—A Contemporary Overview of Acute Coronary Syndrome

**DOI:** 10.3390/jcm13237331

**Published:** 2024-12-02

**Authors:** Alexander Fardman, Fernando Chernomordik, Roy Beigel

**Affiliations:** Cardiovascular Division, Sheba Medical Center, Tel-Hashomer, Ramat-Gan 5262000, Israel; fernando.chernomordik@sheba.health.gov.il (F.C.); roy.beigel@sheba.health.gov.il (R.B.)

Cardiovascular disease (CVD) remains the most common cause of morbidity and mortality worldwide [1]. Acute coronary syndrome (ACS) is frequently the first manifestation of CVD, with an estimated incidence of 7 million new annual cases worldwide [2]. During recent years, there have been advances in the diagnosis and management of ACS. This Special Issue thus comprises publications covering various topics related to the diagnosis and treatment of ACS, highlighting novel advances in the field.

The epidemiology of ACS has changed during recent decades, with a gradual and consistent reduction in ACS incidence in high-income countries, likely due to a higher awareness of and improved care of modifiable risk factors [3]. Although the rupture of lipid-laden plaque remains the most common underlying pathophysiology of ACS, additional mechanisms such as plaque erosions and calcified nodules are more frequently being recognized due to substantial development of intracoronary imaging modalities [4]. Additionally, the presence of myocardial infarction with non-obstructive coronary artery disease (MINOCA) is growing, compromising about 5–6% of patients presenting with an ACS. MINOCA is seen more commonly in younger patients, mainly women, and non-Caucasian ethnicities. The traditional cardiovascular risk factors are less frequently observed in patients presenting with MINOCA [5]. In the current Issue, Caffe A and colleagues discuss different pathophysiological pathways that lead to ACS, including MINOCA, and provide insights into interventions tailored to the underlying mechanisms present [6]. 

The initial presentation of patients with ACS varies from those with subtle chest pain to patients who present with cardiogenic shock and thus require mechanical circulatory support. The initial evaluation and risk stratification of the ACS patient is therefore of paramount importance. Patients presenting with clinically suspected ACS should be immediately evaluated by a resting electrocardiogram (ECG) that will help guide further work up and intervention. Furthermore, an ECG should be performed and interpreted by a qualified physician within 10 min [1]. Patients with ST-segment elevation (STE) should undergo emergent coronary angiography to confirm their diagnosis and perform percutaneous coronary intervention (PCI). In case primary PCI cannot be performed in a timely manner (within 120 min of the first medical contact), a bolus of fibrinolytics should be administered within 10 min of STEMI diagnosis. In patients without STE, the measurement of biomarkers of myocardial injury, preferably high-sensitivity cardiac troponin (hs-cTn), is recommended. Repeated measurements of hs-cTn are the cornerstone of an efficient triage algorithm for patients presenting with a suspected ACS without STE. According to the absolute values and observed changes in hs-cTn, ACS can either safely be “ruled-out” (with a negative predictive value exceeding 99%) or efficiently “ruled-in” in the majority of cases. For patients that do not qualify for either of these categories and have a low to intermediate likelihood of an ACS, contemporary non-invasive imaging with computed tomography angiography (CTA) can be used for ruling out or ruling in the presence of coronary artery disease. In addition to its high negative predictive value in ruling out ACS, CTA can diagnose or rule out life-threatening alternative diagnoses, such as pulmonary embolism or aortic dissection [1].

In patients with non-ST elevation MI (NSTE-MI), the required therapeutic strategy depends on the initial presentation. An immediate invasive approach, such as coronary angiography and PCI (if indicated), is recommended for patients possessing high-risk features, including hemodynamic instability, ongoing refractory chest pain, acute heart failure secondary to myocardial ischemia, life-threatening arrhythmia, mechanical complications, or dynamic ECG changes suggestive of myocardial ischemia. An early invasive strategy, featuring a routine coronary angiography within 24 h of presentation, should be applied in patients exhibiting high-risk criteria: a confirmed diagnosis of NSTE-MI, dynamic ST-segment or T-wave change, transient STE, or a Global Registry of Acute Coronary Events (GRACE) score > 140. In patients without very high- or high-risk features and with a low suspicion of NSTE-MI, an initial non-invasive evaluation with coronary CTA or functional ischemia testing is preferred [1]. 

Antiplatelet treatment remains the cornerstone of medical therapy for ACS patients and includes aspirin and a P2Y12 inhibitor. A potent P2Y12 inhibitor (prasugrel or ticagrelor) is preferred over clopidogrel as the P2Y12 of choice. However, clopidogrel may be considered for older patients, especially for those with a high bleeding risk still in need of dual antiplatelet therapy (DAPT). The Intracoronary Stenting and Antithrombotic Regimen Rapid Early Action for Coronary Treatment (ISAR-REACT-5) trial has shown that a prasugrel-based treatment strategy resulted in a significant reduction in the composite endpoint of death, MI, or stroke (6.9% vs. 9.3%, *p*-value = 0.006) without an increase in bleeding risk (4.8% vs. 5.4%, *p* value = 0.46), as compared with a ticagrelor-based treatment approach [7]. Consequently, current guidelines suggest prasugrel treatment in preference to ticagrelor for patients undergoing an invasive therapeutic approach [1]. The recent European Society of Cardiology guidelines advocated against the routine administration of P2Y12 pretreatment for patients with NSTE-MI treated with an early invasive strategy [1]. This recommendation is mainly based on the lack of benefit with prasugrel pretreatment observed in the ACCOAST trial—A Comparison of Prasugrel at the Time of Percutaneous Coronary Intervention or as Pretreatment at the Time of Diagnosis in Patients with Non-ST Elevation Myocardial Infarction (ACCOAST). It is also based on the inferior results of ticagrelor-based pretreatment approaches as compared to the prasugrel-based strategy, as observed in the ISAR-REACT 5 study [7,8]. The routine administration of intravenous glycoprotein IIb/IIIa inhibitors concomitantly with DAPT is not recommended at present due to the lack of additional benefits as well as an increased bleeding risk. This class of medication should be reserved for bailout therapy in case of thrombotic complications or angiographic evidence of no reflow. 

The third component of antithrombotic therapy for ACS patients is anticoagulation. In patients with STEMI, the peri-procedural administration of unfractionated heparin (UFH) is an established standard of care. In patients with NSTE-MI, the drug of choice depends on the timing of invasive strategies. In those who are treated with an early invasive approach, UFH—or enoxaparin as an alternative—is recommended. However, for patients who do not undergo angiography during the first 24 h, fondaparinux is the preferred medication, with enoxaparin considered as an alternative [1]. Currently, inhibitors of Factor XI are under investigation as a novel approach to further reduce residual thrombotic risk in patients following an ACS event [9].

Approximately half of ACS patients are diagnosed with multivessel coronary artery disease. The timing of the revascularization of non-culprit lesions depends on clinical presentation. The Complete vs. Culprit-only Revascularization to Treat Multivessel Disease After Early PCI for STEMI trial showed that at a median follow-up of 3 years, death from cardiovascular causes or new MI occurred in 7.8% of patients in the complete revascularization group vs. in 10.5% of patients in the group in which only the culprit lesion was treated (hazard ratio [HR], 0.74 [95% CI, 0.60–0.91]; *p*  =  0.004) [10]. Interestingly, the trial did not detect any difference between complete revascularization during the index hospitalization or shortly (within 45 days) after discharge. As such, in stable patients with STEMI, complete revascularization is recommended either during the index hospitalization or within 45 days. Data regarding complete revascularization in patients with NSTEMI is less robust. There is currently no dedicated trial comparing culprit-only versus non-culprit lesion PCI for this patient population. A meta-analysis of 15 non-randomized studies found a lower rate of cardiovascular events and deaths with simultaneous complete revascularization compared to with the revascularization of the culprit lesion alone [11]. As such, a gap in evidence remains regarding the preferred revascularization strategy for stable NSTE-MI patients. Jobs A et al. review different revascularization strategies and available evidence in the current journal issue [12]. 

ACS remains the most common cause of out-of-hospital cardiac arrest (OHCA), although only a minority of patients with ACS present with OHCA. The timing of coronary angiography in patients with OHCA and return of spontaneous circulation (ROSC) depends on ECG findings. If STE is detected, immediate angiography and PCI (if indicated) is recommended. However, for patients without STE following ROSC, routine immediate coronary angiography was not found to be superior to a delayed invasive strategy in terms of survival in two randomized controlled trials [13,14]. As such, it seems reasonable to delay invasive coronary evaluation in hemodynamically stable survivals of OHCA without STE. Temperature monitoring and the active prevention of fever (defined as a temperature above 37.7 C) is recommended in order to improve neurological status. 

Cardiogenic shock complicates 4–11% of ACS patients and can be caused secondary to pump failure, acute mitral regurgitation, or an acute ventricular septal defect. For patients presenting in cardiogenic shock, immediate revascularization should be restricted to the culprit-only lesion, based on the results of the Culprit Lesion Only PCI versus Multivessel PCI in Cardiogenic Shock (CULPRIT-SHOCK) trial. The trial showed a significant reduction in all-cause death or renal replacement therapy at day 30 of follow-up (RR 0.83, 95% CI, 0.71–0.96) in culprit-only treated patients [15]. Notably, in patients with an ACS and cardiogenic shock, the utilization of mechanical circulatory support (MCS) to allow adequate end-organ perfusion seems intuitive. However, until recently, scientific evidence regarding the clinical benefit of MCS devices in cardiogenic shock secondary to an ACS was not available. The routine implantation of an intra-aortic balloon pump device was not associated with a significant survival benefit—currently, this should be considered only for patients with mechanical complications [16]. Similarly, a large randomized multicenter trial failed to show a survival benefit of the implantation of veno-arterial extracorporeal life support (va-ECLS) in patients with ACS and cardiogenic shock [17]. Finally, the routine use of a microaxial flow pump in the treatment of patients with STEMI-related cardiogenic shock led to a lower risk of death from any cause at 180 days (hazard ratio 0.74; 95% CI 0.55–0.99, *p*-value = 0.04) than for standard care alone. However, the incidence of a composite of adverse events was higher with the use of the microaxial flow pump (RR 4.74; 95% CI 2.36–9.55) [18] and should also be taken into consideration.

Despite the progress that has been made in revascularization techniques and antithrombotic therapies, the presence of a left ventricular (LV) thrombus remains a relatively common complication of STEMI patients, especially in those with reduced left ventricular function. The diagnosis of an LV thrombus remains challenging due to the low sensitivity of transthoracic echocardiography and the limited availability of cardiac magnetic resonance (CMR) imaging, considered the gold standard. Several risk scores were recently proposed to identify patients at higher risk for thrombus formation [19,20]. The prospective validation of these scores is still required in order to identify their role in routine clinical practice. 

Continued medical therapy following the acute phase of an ACS has also evolved in recent years. Dual antiplatelet treatment is still generally recommended for 12 months, regardless of the intervention strategy. Recently, several alternative approaches, including a shortened DAPT duration (less than 12 months), extended DAPT duration (more than 12 months), modified DAPT regimens (de-escalation to a less potent P2Y12 agent), and even switching to monotherapy with only a potent P2Y12 inhibitor, have been proposed [21,22,23,24,25,26]. These protocols should not be used as a default but rather tailored to specific patients’ characteristics, such as those with an increased bleeding risk or alternatively those with an increased ischemic risk. Lipid-lowering therapy is another pillar of medical therapy for ACS patients and should be initiated as early as possible. High-potency statins remain the cornerstone of lipid-lowering treatment while ezetimibe and proprotein convertase subtilisin/kexin type 9 (PCSK9) inhibitors should be added in patients that do not reach LDL-cholesterol goals [27,28]. Recently, there has been a focus on evaluating the role of inflammation in the development, prognosis, and treatment of ACS. In the current Issue, two studies address the value of the neutrophil-to-lymphocyte count in ACS and the effect of inflammatory markers on the severity of coronary disease in patients with NSTEMI [29,30]. Notably, anti-inflammatory therapy with colchicine showed a significant reduction in the primary composite outcome (comprised of CV death, resuscitated cardiac arrest, MI, stroke, or urgent revascularization) in comparison to placebo [31] and was proposed as an add-on therapy, in particular for patients with recurrent cardiovascular disease despite having had optimal medical therapy. Several other agents targeting inflammatory pathways, including low-dose Interleukin-2 [32] and Interleukin-6 inhibitors [33], are currently being evaluated in clinical trials. Following an ACS episode, all patients should be referred to participate in a comprehensive cardiac rehabilitation program and receive adequate multi-disciplinary counseling regarding lifestyle modifications, including for smoking and alcohol consumption cessation, maintaining a healthy diet, and undertaking physical activity. 

In summary, significant advances have been achieved in the treatment of ACS over the last decade, resulting in improvements in the diagnosis, management, and survival of patients with an ACS. Nevertheless, ACS still remains an important cause of morbidity and mortality worldwide. As such, further research is warranted to improve the residual risk of ACS patients.
